# Difenoconazole Induced Damage of Bovine Mammary Epithelial Cells via ER Stress and Inflammatory Response

**DOI:** 10.3390/cells13201715

**Published:** 2024-10-17

**Authors:** Myoung-Jun Na, Won-Young Lee, Hyun-Jung Park

**Affiliations:** 1 Department of Animal Biotechnology, College of Life Science, Sangji University, Wonju-si 26339, Republic of Korea; 2 Department of Livestock, Korea National University of Agriculture and Fisheries, Jeonju-si 54874, Republic of Korea; leewy81@korea.kr

**Keywords:** difenoconazole, bovine mammary gland, ER stress, inflammation

## Abstract

Difenoconazole (DIF) is a fungicide used to control various fungi. It is absorbed on the surface of different plants and contributes significantly to increased crop production. However, DIF is reported to exhibit toxicity to fungi and to aquatic plants, fish, and mammals, including humans, causing adverse effects. However, research on the impact of DIF on the mammary epithelial cells of herbivorous bovines is limited. DIF-induced damage and accumulation in the mammary glands can have direct and indirect effects on humans. Therefore, we investigated the effects and mechanisms of DIF toxicity in MAC-T cells. The current study revealed that DIF reduces cell viability and proliferation while triggering apoptotic cell death through the upregulation of pro-apoptotic proteins, including cleaved caspase 3 and Bcl-2-associated X protein (BAX), and the downregulation of leukemia type 2 (BCL-2). DIF also induced endoplasmic reticulum (ER) stress by increasing the expression of genes or proteins of Bip/GRP78, protein disulfide isomerase (PDI), activating transcription factor 4 (ATF4), C/EBP homologous protein (CHOP), and endoplasmic reticulum oxidoreductase 1 Alpha (ERO1-Lα). We demonstrated that DIF induces mitochondria-mediated apoptosis in MAC-T cells by activating ER stress pathways. This cellular damage resulted in a significant increase in the expression of inflammatory response genes and proteins, including cyclooxygenase 2 (COX2), transforming growth factor beta 3 (TGFB3), CCAAT enhancer binding protein delta (CEBPD), and iNOS, in DIF-treated groups. In addition, spheroid formation by MAC-T cells was suppressed by DIF treatment. Our findings suggest that DIF exposure in dairy cows may harm mammary gland function and health and may indirectly affect human consumption of milk.

## 1. Introduction

Difenoconazole (DIF) is a triazole fungicide that is widely used to control various fungal diseases in crops [1]. As a member of the azole class, DIF exhibits broad-spectrum activity against diverse plant pathogens, making it the cornerstone of modern agricultural disease management strategies. Its mode of action involves the inhibition of the biosynthesis of ergosterol, a vital component of fungal cell membranes, leading to the disruption of membrane integrity and subsequent fungal growth inhibition [2,3]. The increase in global food demand has led to an increase in the use of pesticides worldwide, resulting in significant negative impacts on ecosystem players [4].

Exposure to DIF tends to have several interconnected effects on both soil and plant health, potentially acting as an environmental pollutant and posing a long-term risk to soil ecology [5,6]. In soils, it commonly leads to a reduction in the diversity of bacterial communities, along with a decrease in the complexity of their networks [7]. Disruption of soil microbes affects nutrient cycling and plant vitality, thereby affecting crop productivity. Exposure to DIF compromised root development and increased reactive oxygen species (ROS) and MDA production in leaf cells [8]. The elevated levels of oxidative stress indicators signify potential damage to cell membranes and other cellular components that affect plant growth and resilience to environmental stressors [9].

DIF can permeate the surrounding aquatic environment, and fish in direct contact with contaminated water can be used to assess its ecotoxicological effects [10,11]. Other studies have reported that DIF may have various effects on the lifecycle of zebrafish [12]. In zebrafish and carp, interference with DNA integrity and immune function has been observed along with adverse effects on the endocrine system and embryonic development [13,14,15].

The toxic effects of DIF have also been observed in rodents, where DIF exposure affects hepatic toxicity, energy metabolism, and immune-related pathways in mice [16]. Moreover, decreased *mucine2* (*MUC2*) expression may affect intestinal barrier function in mice [17]. According to another study, DIF has four isomers, each of which exhibits different effects, such as inducing lipid droplet accumulation or reducing tight junction protein expression in mice [18]. Exposure to DIF at doses of 0, 20, and 40 mg/kg/day for 35 d resulted in an increased testicular organ coefficient, decreased sperm count and testosterone levels, elevated sperm malformation rates, and induced histopathological alterations, potentially leading to testicular damage. [19].

Residual pesticides can be transmitted and accumulated in consumers, such as mammals, including humans, through animal feed and the food chain [20,21]. Confirming its direct impact on humans can be challenging; thus, research focusing on livestock, including cows, has garnered attention. Studies have revealed the presence of pesticides in cattle feed, leading to the detection of pesticide residues in raw cow milk as well [22,23]. In one study, the fungicide tetraconazole was applied to bovine mammary epithelial cells, resulting in the deterioration of milk production due to the disruption of calcium homeostasis and mitochondrial function [24]. In another study, exposure to the herbicide bifenox induced ER stress and cell inflammation, resulting in the death of bovine mammary epithelial cells and decreased casein [25]. Furthermore, bifenthrin, a synthetic pyrethroid insecticide, reduces mitochondrial membrane potential (MMP) and disrupts cell proliferation by generating excess ROS [26].

The accumulation of pesticides in dairy cows has a notable impact on mammary gland conditions and milk production. Despite the toxicity of DIF demonstrated in various fields, its effects on mammals remain unclear. In this study, we investigated the effects of DIF on bovine mammary epithelial cells, commonly referred to as MAC-T cells, and attempted to elucidate the underlying molecular mechanism.

## 2. Materials and Methods

### 2.1. Cell Culture and Chemicals

Bovine mammary epithelial cells (MAC-T) were cultured using Dulbecco’s Modified Eagle’s Medium (DMEM) supplemented with 10% (*v*/*v*) heat-inactivated FBS, 100 U/mL penicillin sodium, and 100 μg/mL streptomycin solution. The cells were maintained in a 5% CO_2_ atmosphere at 37 °C. Difenoconazole was purchased from Sigma-Aldrich (St. Louis, MO, USA) and dissolved in dimethyl sulfoxide (DMSO) to make a 1 M stock, which was diluted to the final concentration used in these experiments.

### 2.2. Cell Viability Assay

The 3-(4,5-dimethylthiazole-2-yl)-2,5-diphenyl tetrazolium bromide (MTT) assay is a colorimetric assay that determines cell viability. The EZ-Cytox Viability Assay Kit (Daeil Lab Services Co., Seoul, Republic of Korea, #EZ1000) was used to evaluate DIF cytotoxicity in MAC-T cells. First, a cell suspension was prepared at a concentration of 1 × 10^5^/mL, and 2 × 10^4^ cells were seeded in each well of a 6-well plate along with the culture medium. After culturing for 12–14 h, each well was treated with DIF concentrations of 0, 10, 50, 100, 500, and 1000 μM, followed by an additional 24 h incubation period. Subsequently, according to the manufacturer’s instructions, 10% of the total volume of each well was supplemented with the EZ-Cytox reagent and incubated for 1 h incubation. The solution was then dispensed into a 96-well plate. Absorbance was measured at 450 nm using an epoch spectrophotometer (Bio Tek, Winooski, VT, USA). After DIF treatment, cell images were obtained under a microscope before the addition of the MTT reagent.

### 2.3. Spheroid Formation and Treatment

MAC-T cells were transduced with enhanced green fluorescent protein (mAct-GFP) using lentiviral gene transfer. The mAct-GFP-infected cells were then counted and diluted to a concentration of 0.5 × 10⁴ before being seeded into a 96-well Corning spheroid microplate (Corning Inc., Philadelphia, PA, USA). Afterward, the cells were treated with Geltrex™ (LDEV-Free Reduced Growth Factor Basement Membrane Matrix) and DIF, and observations were conducted at 24, 48, and 72 h, and images were collected using fluorescent microcopy (Olympus IX73, Olympus, Tokyo, Japan).

### 2.4. Flow Cytometry

To investigate cell death and mitochondrial membrane potential, two different staining methods were used and analyzed using flow cytometry (CytoFLEX, Beckman Coulter, Inc., Miami, FL, USA). First, the presence of apoptosis was determined using the Alexa Fluor 488 Annexin V/Dead Cell Apoptosis Kit (Invitrogen; Thermo Fisher Scientific, Inc., Waltham, MA, USA). MAC-T cells were seeded in a 6-well plate at a density of less than 1 × 10^6^ cells/mL and treated with DIF at concentrations ranging from 0 to 100 µM. Cells were harvested by trypsinization and washed with DPBS. Following the manufacturer’s instructions, cells were stained by mixing annexin V-FITC and propidium iodide (PI) reagents with 1 × annexin-binding buffer. After staining, in the final step, the cells are resuspended in 400 µL of annexin buffer and detected by flow cytometry. Second, changes in MAC-T cells induced by DIF treatment were assessed using the JC-1 Mitochondrial Membrane Potential Detection Assay Kit (Biotium Inc., Fremont, CA, USA). After seeding at a density of less than 1 × 10^6^ cells/mL, similar to the previous staining, the cells were treated with DIF and harvested 24 h later. Following the manufacturer’s instructions, staining was performed using the JC-1 reagent, and in the final step, the cells were resuspended in DPBS before measuring MMP using a flow cytometer. After JC-1 staining, cell images were obtained using a fluorescence microscope (Olympus IX73; Olympus, Tokyo, Japan).

### 2.5. Immunocytochemistry

Immunocytochemistry (ICC) is the process of confirming and visualizing the expression of a desired protein within cells using antibodies that specifically recognize the target protein. In this study, proliferation and inflammation levels were assessed using antibodies, ki67, and COX2 through an ICC experiment. Coverslips were sterilized before seeding the cells onto 18 mm glass coverslips (BD Biosciences, Franklin Lakes, NJ, USA). Sterilization was achieved by dipping the samples in ethanol followed by flaming. Afterward, MAC-T cells were cultured on coverslips for approximately 8 h, followed by treatment with various concentrations (0, 10, 50, and 100 μM) of DIF for 24 h. To preserve and maintain the cell structure, a fixation step was performed using 4% paraformaldehyde at room temperature for 15 min. Following fixation, the cells were washed with PBS, and antibody treatment and data analysis were conducted as previously described [27]. The antibodies used are listed in Table 1. Antibodies were purchased from Santa Cruz Biotech (Dallas, TX, USA), Abcam (Cambridge, UK), and Cell signaling Technologies (Danvers, MA, USA). 

### 2.6. Quantitative PCR (QPCR)

Cells were treated with DIF for 24 h, and total RNA was extracted using the RNeasy Mini Kit (Qiagen, Hilden, Germany) with RNase-free DNase, according to the manufacturer’s instructions. Total pure RNA was extracted. First, template RNA and oligo dT were mixed and incubated at 65 °C for 5 min using a PCR machine. After this incubation, mix the sample with the components of the RevertAid First Strand cDNA Synthesis Kit (Thermo Scientific, Rockford, IL, USA) and incubate using a PCR machine at 42.0 °C for 1 h, followed by 25.0 °C for 5 min and 70.0 °C for 5 min to initiate reverse transcription. The synthesized cDNA was then diluted to a final concentration of 10 ng/µL using pure water. To perform real-time PCR (quantitative PCR, qPCR), cDNA, SYBR Green qPCR Master Mix (Thermo Fisher Scientific, Waltham, MA, USA), primer, and pure water were each dispensed and mixed appropriately under consistent conditions. The reactions were conducted using a QuatStudio 1 instrument (Applied Biosystems, Foster City, CA, USA) with the following cycling conditions: an initial step at 95 °C for 10 min, followed by 40 cycles of 95 °C for 15 s, 57 °C for 10 s, and 72 °C for 20 s. Genetic normalization was performed using the housekeeping gene, *GAPDH*. Table 2 shows the primers used to identify specific genes and their corresponding nucleotide sequences.

### 2.7. Western Blotting

Proteins were harvested using RIPA lysis buffer (Thermo Scientific, Rockford, IL, USA) supplemented with a protease inhibitor mixture (Roche, Rotkreuz, Switzerland). The bicinchoninic acid (BCA) assay was conducted according to the manufacturer’s instructions to determine protein concentration. Subsequently, each 30 μg of protein was loaded into SDS-PAGE gel. The separated proteins were transferred onto polyvinylidene fluoride (PVDF) membranes. Following that, the membranes were incubated overnight at 4 °C with primary antibodies in a solution containing 1% BSA in TBS-Tween-20 (0.05% Tween-20). The membranes were then incubated with anti-mouse or anti-rabbit secondary antibodies for 1 h depending on the type of primary antibody used. Afterward, the cells were washed with TBS-T for 2 h. ECL solution (Cytiva, Marlborough, MA, USA) was applied to the membranes and visualized using iBright™ Imaging Systems (Thermo Fisher Scientific, Inc., Waltham, MA, USA). β-actin was used as the normalization control, and the antibodies used for immunoblotting are listed in Table 1.

### 2.8. Statistical Analysis

Data were expressed as the mean ± standard error (SEM) of at least three independent experiments conducted in triplicate. Mean differences were evaluated using one-way analysis of variance (ANOVA), followed by Tukey’s post hoc test. All statistical analyses were conducted using the SPSS statistical package, version 15.0, for Windows (IBM Corp, Somers, NY, USA). Comparisons were considered statistically significant at * p < 0.05, ** p < 0.01, and *** p < 0.001.

## 3. Results

### 3.1. DIF Induced MAC-T Cell Toxicity and Anti-Proliferation

**Figure 1:**
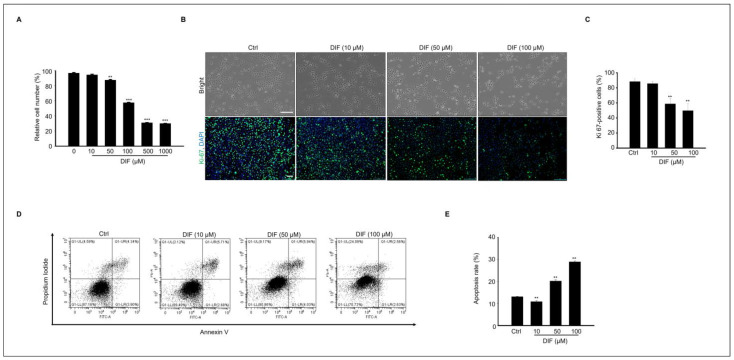
Effects of DIF on MAC-T cells include significant impacts on both cell apoptosis and proliferation. (**A**) MAC-T cell viability through an MTT assay. Cells were treated with DIF (0–1000 µM). Data represent the mean ± SD (n = 5, ** *p* < 0.01, *** *p* < 0.001 compared to the control). (**B**) After treating cells with DIF (0–100 μM) followed by a 24 h incubation period, the morphology of the cells was observed using a microscope, and immunostaining was performed using Ki-67 antibody. Scaler bar = 100 μM. (**C**) The graph illustrating the ratio of Ki-67-positive cells to the total cells stained with DAPI has been created for the manuscript (n = 4, ** *p* < 0.001). (**D**) After culturing MAC-T cells with DIF at concentrations ranging from 0 to 100 μM, annexin V-FITC/PI staining was conducted. Following staining, dye-positive/negative cells were assessed using flow cytometry to determine cell death by apoptosis. (**E**) The graph depicts the proportion of apoptosis as mean ± SD based on the results of flow cytometry analysis. (n = 4, ** *p* < 0.001).

The cytotoxicity of DIF on MAC-T cells was examined by assessing cell viability using an MTT assay. Cells were treated with various concentrations (0–1000 μM) of DIF for 24 h (Figure 1A). No significant decrease was observed in cell viability observed up to 50 μM; however, a sharp decline was noted beyond 50 μM. Additionally, when observed under a microscope, no morphological changes were detected at 10 μM, whereas distinct alterations were observed from a concentration of 50 μM onward (Figure 1B). Negative impact of DIF on MAC-T cell viability. Immunocytochemistry using Ki-67 antibody was conducted to investigate the effect on cell proliferation. The result showed that the percentage of Ki-67-positive cells was significantly decreased in 50–100 μM DIF-treated groups. Based on these results, to assess the impact of DIF on MAC-T cell death, annexin V staining was conducted, and confirmation was performed via flow cytometry. Through the staining patterns of FITC-conjugated annexin V and propidium iodide (PI), late apoptotic cells (annexin V-FITC+/PI+) and necrotic cells (annexin V-FITC-/PI+) were identified (Figure 1D). Our findings reveal that approximately 30–35% of the cells underwent apoptosis following treatment with 100 μM DIF (Figure 1E). In Figure 1, our results confirmed that from 50 μM onward, DIF has a significant impact on MAC-T cell proliferation and cell death.

### 3.2. DIF Induces Apoptosis and Mitochondrial Dysfunction in MAC-T Cells

**Figure 2:**
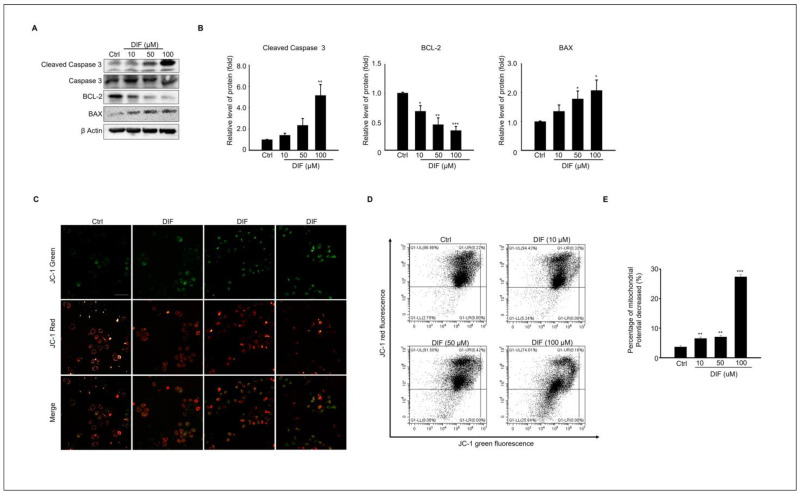
Expression levels of Pro-apoptotic protein, and mitochondrial dysfunction in DIF exposed MAC-T cell. (**A**) MAC-T cells were treated with DIF concentrations ranging from 0 to 100 μM for 24 h, followed by preparation of total protein and analysis via immunoblotting. The protein expression levels of cleaved caspase 3, caspase 3 and BAX, Bcl-2, and β-actin were evaluated in each experimental group. (**B**) The graphs display protein bands represented as quantified data normalized to β-actin as mean ± SD (n = 5, * *p* < 0.05, ** *p* < 0.01, *** *p* < 0.001). (**C**) Cells treated with DIF (0–100 μM) were cultured in a 6-well plate, followed by JC-1 staining. Red and green fluorescence were observed using fluorescence microscopy. The ratio of JC-1 green emitted at 530 nm and JC-1 red emitted at 580 nm varies depending on the change in mitochondrial membrane potential. Scale bar =100 μm. (**D**) Flow cytometry analysis was conducted for quantification of JC-1 green and red. (**E**) A graph was generated based on the decrease in the proportion predicted to be JC-1 red fluorescence (% J-monomers/J-aggregates), and data show mean ± SD. (n = 5, ** *p* < 0.01, and *** *p* < 0.001).

We evaluated whether DIF regulates the expression of pro-apoptotic proteins in cultured MAC-T cells. The protein expression of cleaved caspases 3, BAX, and Bcl-2, which are pro-apoptotic proteins, was examined (Figure 2A). The results showed that the expression levels of cleaved caspases 3 and BAX increased, whereas the expression level of Bcl-2 protein decreased in a dose-dependent manner (Figure 2D). Mitochondria is a crucial player in activating apoptosis in various cell types [28]. Therefore, mitochondrial dysfunction was assessed using JC-1 staining. DIF treatment followed by JC-1 staining of MAC-T cells allowed the assessment of mitochondrial dysfunction by measuring mitochondrial membrane potential (MMP). JC-1 staining in its polymeric form indicates a high mitochondrial membrane potential by emitting red fluorescence, whereas in its monomeric form, it indicates a low membrane potential by emitting green fluorescence. The image shows a reduction in both the red fluorescence intensity and the Q1-UL region with increasing concentrations of DIF. These observations suggest that DIF lowers the mitochondrial membrane potential in MAC-T cells, indicating mitochondrial dysfunction (Figure 2D). The ratio of red-to-green fluorescence was normalized to that of the control. Notably, the relative MMP ratio exhibited a dose-dependent decline under DIF treatment, particularly evident at concentrations ranging from 50 to 100 μM (Figure 2E).

### 3.3. Endoplasmic Reticulum (ER) Stress-Mediated MAC-T Cell Death Following DIF Treatment

**Figure 3:**
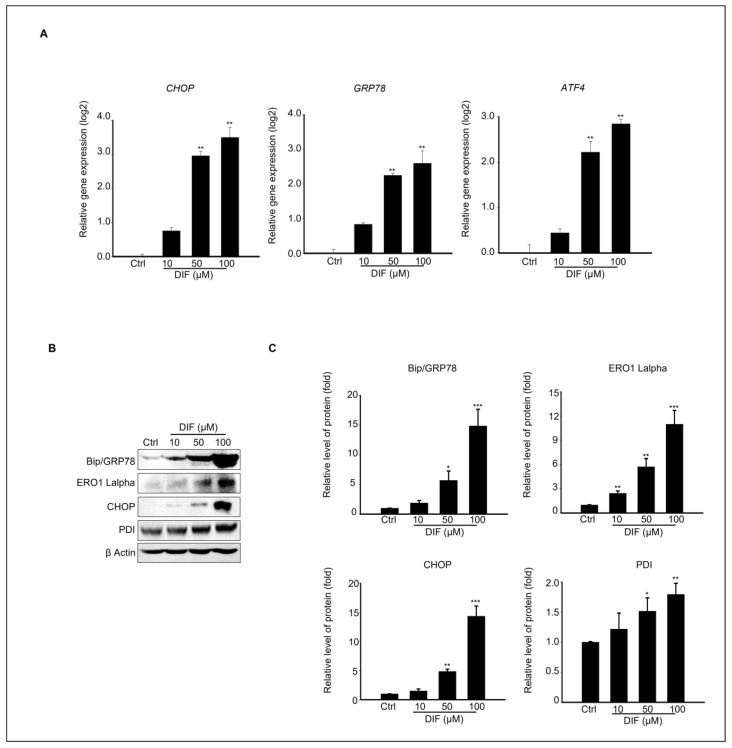
Effects of DIF on ER stress in MAC-T cell. (**A**) qPCR analysis of CHOP, Bip/Grp78, and ATF4 expression levels following 24 h of DIF treatment. Data represent mean ± SD with log2 scales (n = 5, ** *p* < 0.01 compared to controls). (**B**) Immunoblot analysis showing the protein levels of BiP/GRP78, ERO1-Lα, CHOP, PDI, and β-actin after DIF exposure. (**C**) Densitometric quantification of protein bands normalized to β-actin. Results are presented as the mean ± SD (n = 5, * *p* < 0.05, ** *p* < 0.01, *** *p* < 0.001).

ER stress contributes to apoptosis by regulating various molecules, including CHOP, GRP78, and ATF4 [29]. Therefore, we investigated the mechanisms underlying ER stress by treating MAC-T cells with DIF. The gene and protein expression levels of ER stress-signaling molecules were evaluated in cells exposed to 0–100 μM DIF. These findings suggest that DIF induces significant upregulation of transcriptional levels of CHOP and GRP78 at the highest concentration (100 μM), whereas ATF exhibited the greatest increase at DIF 50–100 μM (Figure 3A). Consistent with the gene expression results, DIF treatment upregulated the ER stress-related protein levels (Figure 3B). Key ER stress-related protein levels such as BiP/GRP78, CHOP, PDI, and ERO1-Lα were statistically elevated in 50–100 μM DIF-treated samples compared to those of the control in a dose-dependent manner (Figure 3C).

### 3.4. DIF Induces Inflammatory Responses in MAC-T Cells

**Figure 4:**
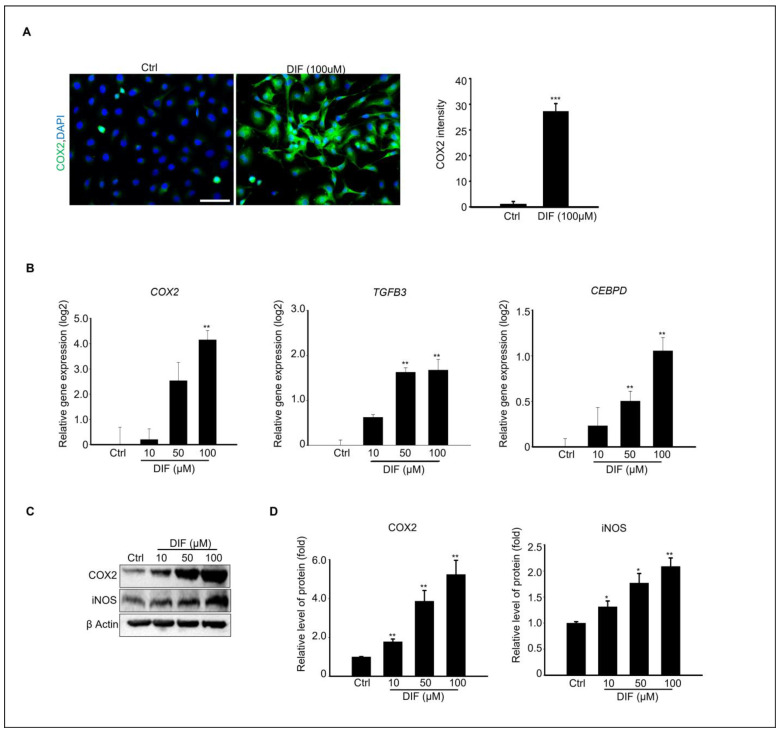
DIF induced inflammation at cultured MAC-T cell. (**A**) Immunostaining of COX2 protein in100 μM DIF treated MAC-T cells. Scale bar = 50 μM. DAPI were stained nucleus. Graph showed COX2 intensity from each group. *** *p* < 0.001, (**B**) The relative gene expression of *Cox2, TGFB2*, and *CEBPD* in each group after 0–100 μM DIF treatment for 24 h. Graph showed mean ± SD (n = 5, ** *p* < 0.001 compared to controls). (**C**) Protein expression of COX2, iNOS, and β-actin after 0–100 μM DIF exposure. (**D**) Graph represents each protein band normalized to β-actin as mean ± SD (n = 5, * *p* < 0.05, ** *p* < 0.01 compared to controls).

Various studies have shown that inflammatory cytokines can induce ER stress, leading to the activation of the unfolded protein response (UPR). For instance, tumor necrosis factor-alpha (TNF-α) causes ER stress, activating PERK, IRE1 α, and ATF6 in fibrosarcoma cells [30]. Based on these findings, we investigated whether DIF causes inflammation in MAC-T cells. To confirm this relationship, immunocytochemistry was performed using COX2 antibody. Microscopic examination revealed a significant increase in COX2 expression compared to the control, supporting the association between DIF exposure and inflammation (Figure 4A). The gene expression levels of inflammation-regulating genes, *Cyclooxygenase 2* (*Cox2*), *Transforming growth factor beta-3* (*Tgfβ-3*), and *CEBPD CCAAT*/*enhancer binding protein* (*C*/*EBP*), were found to be significantly increased, as confirmed by qPCR (Figure 4B). Immunoblotting was performed to examine the expression levels of the inflammatory proteins COX2 and iNOS in response to increasing concentrations of DIF (Figure 4C). A gradual increase in protein expression was observed, confirming an increase in the levels of inflammatory markers. (Figure 4D). This pattern of increased gene expression is consistent with the observed increase in protein expression.

### 3.5. DIF Treatment Effects on Spheroid Culture of MAC-T Cells

**Figure 5:**
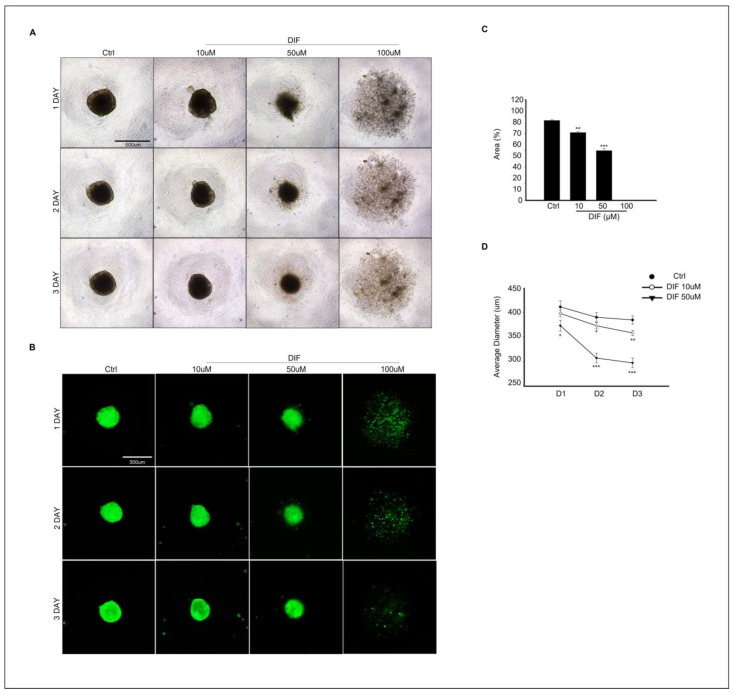
Effects of DIF on spheroid formation from MAC-T cells. (**A**) Morphological change in spheroid from MAC-T cell after 0–100 μM DIF treatment for 3 d. Images were captured using bright-field microscopy, with the scale bar representing = 500 μM. (**B**) GFP expressing spheroid of MAC-T cell (green fluorescent) after 0–100 DIF μM treatment. Scale bar = 500 μM. (**C**) The area of spheroid (%) from each group. Graph showed mean ± SD (** *p* < 0.01, *** *p* < 0.001 compared to controls). Spheroid formation did not occur in 100 μM DIF-treated groups. (**D**) Average diameter (μm) of spheroid from each group. The diameter measurement was carried out from D1–D3. Graph showed mean ± SD (* *p* < 0.05, ** *p* < 0.01, *** *p* < 0.001 compared to controls).

MAC-T cell spheroid formation was evaluated at different concentrations of DIF treatment in culture. In Figure 5A, the image of the control group shows that the spheroid was well formed after 24 h of culture, and the shape was maintained until the 3rd day of culture. However, as the treatment concentration of DIF increases, the spheroid size decreases in cells exposed to 10–50 μM DIF or fails the spheroid formation at 100 μM DIF treatment (Figure 5A). To identify the morphological changes in spheroids more clearly, spheroid formation was induced in the same manner as in Figure 5A using MAC-T cells expressing green fluorescent genes, and images were collected (Figure 5B). For quantitative analysis, the area (%) and diameter of the spheroids were analyzed (Figure 5C,D). The area of spheroid (%) was significantly decreased in 10–50 μM DIF treatment in a dose-dependent manner, and spheroid formation did not occur in 100 μM DIF-treated groups (Figure 5C). The average diameter of spheroid significantly decreased in the 10–50 μM DIF-treated groups after 24 h of culture. Particularly, the area of spheroid decreased by 25% in the 50 μM treatment groups compared to that of the control groups (Figure 5D).

## 4. Discussion

Difenoconazole is a triazole fungicide widely used to eliminate pests and increase crop productivity. However, its use has resulted in its detection in soil and groundwater. It can also leach into marine and freshwater environments, thereby affecting numerous aquatic plants and animals within these ecosystems [31]. Additionally, difenoconazole can bioaccumulate in some aquatic organisms and can then be magnified through the food chain from phytoplankton to fish, birds, and mammals. Therefore, it is crucial to elucidate the molecular mechanisms underlying the toxic effects of difenoconazole.

Previous studies demonstrated that the fungicide tebuconazole induces ER stress in MAC-T cells [32]. In addition, aclonifen suppressed the proliferation of MAC-T cells, induced apoptotic cell death via ROS production, and interrupted intracellular calcium homeostasis. Similar to our results, aconifen promotes the expression of inflammation-related genes, such as PTGS2 and CXCL8 [33]. According to our result, a toxicological evaluation of DIF on MAC-T cells revealed that it significantly impacts cell viability in a concentration-dependent manner. Cell death can occur for various reasons. It is programmed to remove unnecessary parts during tissue development, defend against external antigens, and maintain homeostasis [34]. This process can be categorized into three forms: apoptosis, necrosis, and autophagy. These forms are activated through specific signaling pathways and can occur independently or are interrelated [35]. Apoptosis is divided into extrinsic and intrinsic signaling pathways, each characterized by specific proteins through which their respective pathways can be identified [36]. In this study, we found that Bcl-2 expression decreased, whereas BAX and cleaved caspase 3 levels increased. Furthermore, as the concentration of DIF increased, these changes became more pronounced. The Bcl-2 family serves as a key regulator of the intrinsic apoptotic pathway, where one of its members, Bcl-2, functions as an anti-apoptotic protein, and Bax operates as a pro-apoptotic protein [37].

Additionally, Bcl-2 is located on the outer mitochondrial membrane and regulates mitochondrial outer membrane permeability (MOMP), preventing the release of activators of the caspase cascade [38,39]. However, the expression of cleaved caspase 3 increased and that of Bcl-2 protein decreased, indicating potential dysfunction in the mitochondria. To assess this, we measured mitochondrial membrane potential (MMP) as an indicator of mitochondrial function and confirmed a decrease. MMP is generated by the redox reactions of electron transport chain complexes located in the inner mitochondrial membrane, leading to the formation of a proton gradient [40], which is involved in energy conversion, enabling normal physiological activities and influencing mitochondrial homeostasis to ensure its maintenance [41]. This decrease in MMP leads to a reduction in ATP production, preventing cells from obtaining the required energy. This can induce cellular dysfunction and death [42,43].

In our results, ER stress was increased by DIF treatment. Immunoblotting results revealed that treatment with 100 μM DIF led to an increase in the expression of ER stress-related proteins, including Bip/GRP78, ERO1 Lalpha, CHOP, and PDI, with levels rising by at least 2-fold and up to 16-fold. Additionally, gene expression analysis showed that CHOP and Bip/GRP78 exhibited a similar pattern of increase and that ATF4 expression was also elevated. CHOP is transcriptionally activated in response to cellular stress signals, such as heat shock, UPR, and DNA damage. When ATF4 levels increase, it binds to the Gadd153 (CHOP) promoter, promoting its expression [44,45]. Several studies reported that ER stress induces the activation of cell death [46,47]. The endoplasmic reticulum (ER) processes membranes and secreted proteins to ensure that they are properly functional and stores free calcium to release it when needed, maintaining cellular homeostasis through appropriate physiological regulation [48]. However, various factors, such as fungicide accumulation, can disrupt ER function, leading to an increase in misfolded and unfolded proteins, excessive Ca^2+^ release, and Ca^2+^ depletion within the ER [49,50,51]. This disruption results in a failure to maintain homeostasis, ultimately triggering apoptosis [51,52]. When ER stress occurs, the cell activates a complex signaling pathway known as the unfolded protein response (UPR) to recover and alleviate stress [53]. This mechanism helps maintain homeostasis by addressing and resolving protein folding issues. If the UPR is activated but fails to resolve this issue, ER stress can trigger either intrinsic pathways related to mitochondria or extrinsic pathways involving death receptors, leading to cellular dysfunction or apoptosis [54,55].

Chiu et al. described the effect of carvacrol, a monoterpernoid phenol, which has antimicrobial and anti-inflammatory activities, on the osteosarcoma cell line. The results showed that induced ER stress can protect cells against apoptosis; elevated ROS levels promote apoptosis in carvacrol-treated cells. Although carvacrol is not a pesticide, its correlation with ER stress and apoptosis is different from our study [56].

Mammary gland epithelial cells (MECs) perform crucial roles, including providing nutrients to offspring, transferring immunity from the mother to the newborn, and defending against infections. This defense mechanism involves collaboration between MECs and various immune cells. The mammary glands secrete nutrient-rich milk that can support bacterial growth and shape its response to bacterial invasion [57]. Therefore, damage to the MECs can severely affect milk production. Although our results were limited to in vitro studies, we could not establish a correlation between the immune cells and external bacteria. However, our results showed that DIF alone increased the expression of inflammation-related genes and proteins in MAC-T MECs. The protein expression levels of inflammatory markers, such as COX-2 and iNOS, as well as the mRNA levels of Tgfb3, CEBPD, and Cox-2, were increased by DIF in our study. COX-2 is involved in the production of prostaglandins, and together with iNOS, it modulates inflammation through the NF-κB signaling pathway, which can either upregulate or downregulate the inflammatory response [58,59]. CEBPD binds to specific regulatory regions of the COX-2 promoter to modulate its transcriptional activation, thereby promoting the expression of COX-2 [60]. Various studies have reported a correlation between the inflammatory response and apoptosis. Necrosis triggers an inflammatory response, whereas apoptotic cell death is generally considered non-inflammatory [61,62]. In contrast, Haanen et al. have demonstrated that apoptotic cell death is crucial for triggering and resolving the inflammatory response [63], which supports our results.

Although bovine mammalian epithelial cells, which typically grow in a monolayer, have been used, this approach does not fully replicate the physiological conditions. In epithelial tissues, cells connect via cell junctions and engage in complex interactions. To investigate how DIF treatment affects cell–cell adhesion, 3D cell culture was performed alongside DIF treatment. Spheroid formation from MAC-T cells showed that DIF decreased the relative area and diameter of the spheroids after 72 h of culture. This is the first time that 3D spheroids have been formed using a polystyrene round-bottomed microwell and a Geltrex^TM^ matrix. It was confirmed that spheroid formed successfully, but no formation occurred at the highest (100 μM) concentration of DIF treatment.

In conclusion, the current study underscores the potential risks of DIF exposure in dairy cattle by exploring its toxic effect on MAC-T cells, a type of MEC. DIF led to a decrease in viable MAC-T cells, mirroring the results in spheroid cultures, and impaired mitochondrial membrane potential, ER stress, and subsequent apoptosis. DIF also induces the expression of inflammation-related genes in MAC-T cells. However, the evaluation of the impact of DIF in an in vitro system is limited. Understanding these cellular responses is crucial for indirectly assessing the impact of toxicants such as DIF on milk production, and this is the first study to suggest that DIF poses a risk to MECs. Milk production quantity and quality are crucial not only for farmers’ income but also for providing healthy food to consumers.

## Figures and Tables

**Table 1:** List of antibodies for immunostaining and blotting.

Antibody	Manufacturer	Catalog Number	Dilution (WB)	Dilution (ICC)
β-actin	Santa Cruz Biotech	SC-47778	1:1000	
Bcl-2	Cell Signaling	15071S	1:2000	
Cleaved-caspase 3	Cell Signaling	9661S	1:2000	
Caspase 3	Santa Cruz	SC-22171	1:2000	
BAX	Santa Cruz	SC-7480	1:2000	
BIP/GRP78	Cell Signaling	3177T	1:2000	
ERO-La	Cell Signaling	3264T	1:2000	
PDI	Cell Signaling	3501S	1:2000	
CHOP	Cell Signaling	2895S	1:2000	
iNOS	Cell Signaling	13120	1:2000	
COX2	Cell Signaling	12282	1:2000	1:200
Ki 67	Abcam	Ab15580		1:200

**Table 2:** List of primer for quantitative PCR (QPCR).

Gene	Forward Primer	Reverse Primer
*GAPDH*	5′-GGGTCATCATCTCTGCACCT-3′	5′-GGTCATAAGTCCCTCCACGA-3′
*COX2*	5′-TCCTGAAACCCACTCCCAACA-3′	5′-TGGGCAGTCATCAGGCACAG-3′
*Tgfb3*	5′-TCTGGGGCGACTTAAGAAGA-3′	5′-ATTGCGGAAGCAGTAATTGG-3′
*CEBPD*	5′-ATCGACTTCAGCGCCTACAT-3′	5′-TGTGGTTGCTGTTGAAGAGG-3′
*CHOP*	5′-GCAACGCATGAAGGAGAAAG-3′	5′-AACCATCCGGTCAATCAGAG-3′
*Grp78*	5′-TGGCTGGAAAGTCACCAAG-3′	5′-GTCTGCTGCTTCCTCCTCAC-3′
*ATF4*	5′-GCTGTGGATTGGTTGGTCTC-3′	5′-AGCTCATCTGGCATGGTTTC-3′
*Nrf2*	5′-AGGACATGGATTTGATTGAC-3′	5′-TACCTGGGAGTAGTTGGCA-3′
*HO-1*	5′-GGTGATGGCGTCTTTGTACC-3′	5′-GCAGCTCCTCTGGGAAGTAG-3′

## Data Availability

Data will be made available on request.
